# Supplementary material for: Astrocytic metabolic control of orexinergic activity in the lateral hypothalamus regulates sleep and wake architecture
Source: Nat Commun. 2024 Jul 16;15:5979. doi: 10.1038/s41467-024-50166-7 (PMC11252394; doi:10.1038/s41467-024-50166-7)
Supplement: Supplementary file 1 — Supplementary Information [file 41467_2024_50166_MOESM1_ESM.docx]

Astrocytic metabolic control of orexinergic activity in the lateral hypothalamus regulates sleep and wake architecture

Alice Braga^1*^, Martina Chiacchiaretta^1*^, Luc Pellerin^2^, Dong Kong^1,3^, Philip G. Haydon^1^.

^1^Department of Neuroscience, Tufts University School of Medicine, Boston, Massachusetts, 02111, USA.

^2^Inserm U1313, University and CHU of Poitiers, 86021 Poitiers, France.

^3^Division of Endocrinology, F.M. Kirby Neurobiology Center, Department of Pediatrics, Boston Children's Hospital and Harvard Medical School, Boston, Massachusetts 02115, USA.

*Contributed equally

Corresponding authors:

Martina Chiacchiaretta, Ph.D; e-mail: [martina.chiacchiaretta@tufts.edu](mailto:martina.chiacchiaretta@tufts.edu)

Philip G. Haydon, Ph.D.; e-mail: philip.haydon@tufts.edu


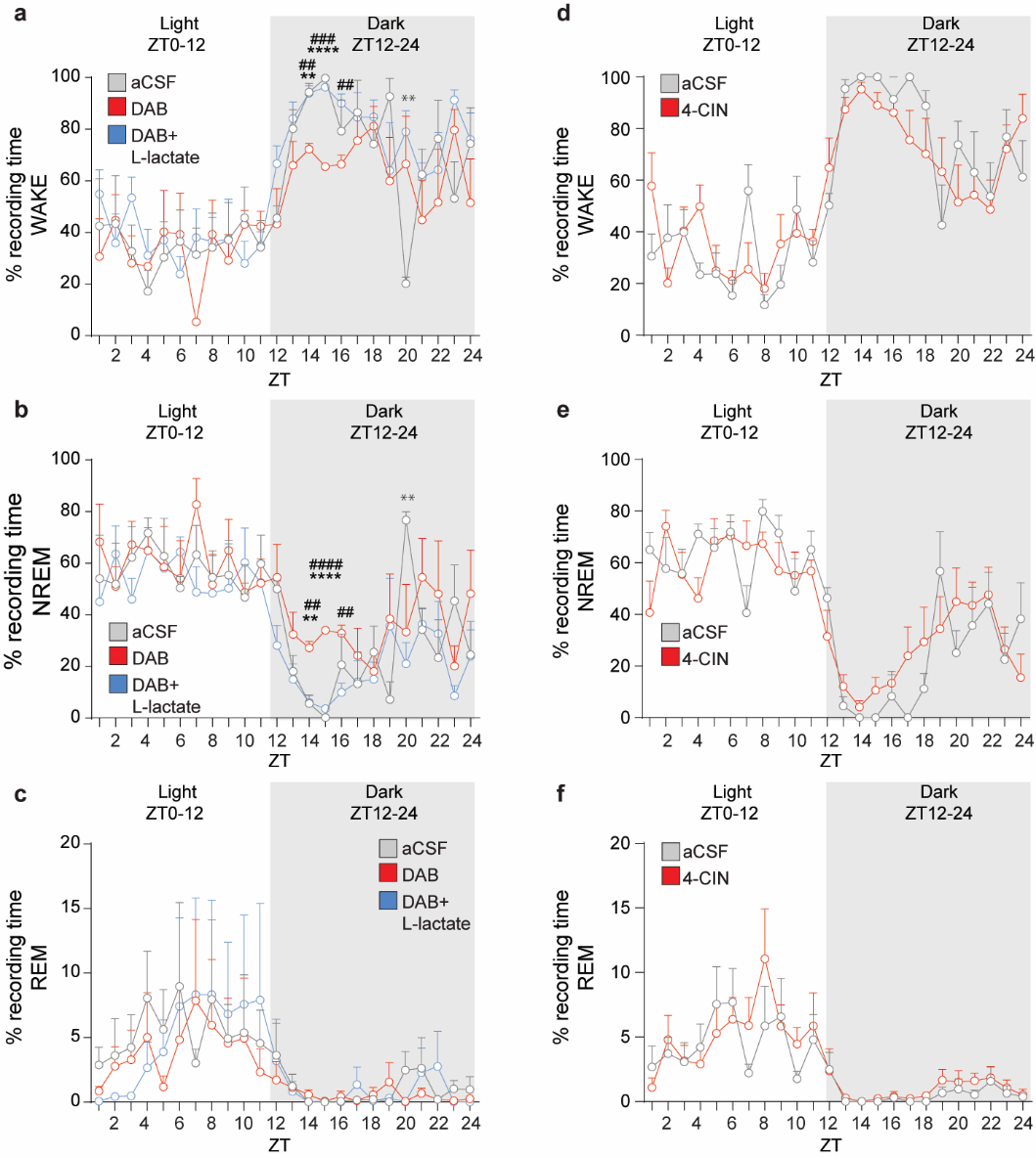


**Supplementary Fig. 1. Sleep/wake pattern in WT mice upon blockage of glycogenolysis or MCTs in the LH**

(a - c) Averaged percentage of the time spent in wake (a), NREM (b), and REM sleep (c) per hour in WT mice upon DAB or DAB+L-lactate infusion compared to control mice.

(d - f) Averaged percentage of the time spent in wake (d), NREM (e), and REM sleep (f) per hour in WT mice upon 4-CIN infusion compared to control mice. (a , b and c: n=4, Two-Way ANOVA followed by Sidak post hoc test; %wake DAB vs DAB+L-lact ##p=0.003, aCSF vs DAB **p=0.006, DAB vs DAB+L-lact ###p=0.0001, aCSF vs DAB ****p<0.0001, DAB vs DAV+L-lact ##p=0.008, aCSF vs DAB+L-lact **p=0.008; %NREM aCSF vs DAB **p=0.007, DAB vs DAB+L-lact ##p=0.003, aCSF vs DAB and DAB vd DAB+L-lact **** p<0.0001, DAB vs DAB+L-lact ##p=0.008, p<0.0001, aCSF vs DAB+L-lact **p=0.008; aCSF infusion in grey, DAB infusion in red, DAB + L-lactate infusion in blue; d, e and f: n=6, Two-Way ANOVA followed by Sidak post hoc test; aCSF infusion in grey, 4-CIN infusion in red). ZT= Zeitgeber Time; DAB=1,4-dideoxy-1,4-imino-d-arabinitol; 4-CIN=alpha-cyano-4-hydroxycinnamate. Pooled data are shown as mean ± SEM. Source data are provided as a Source Data file.


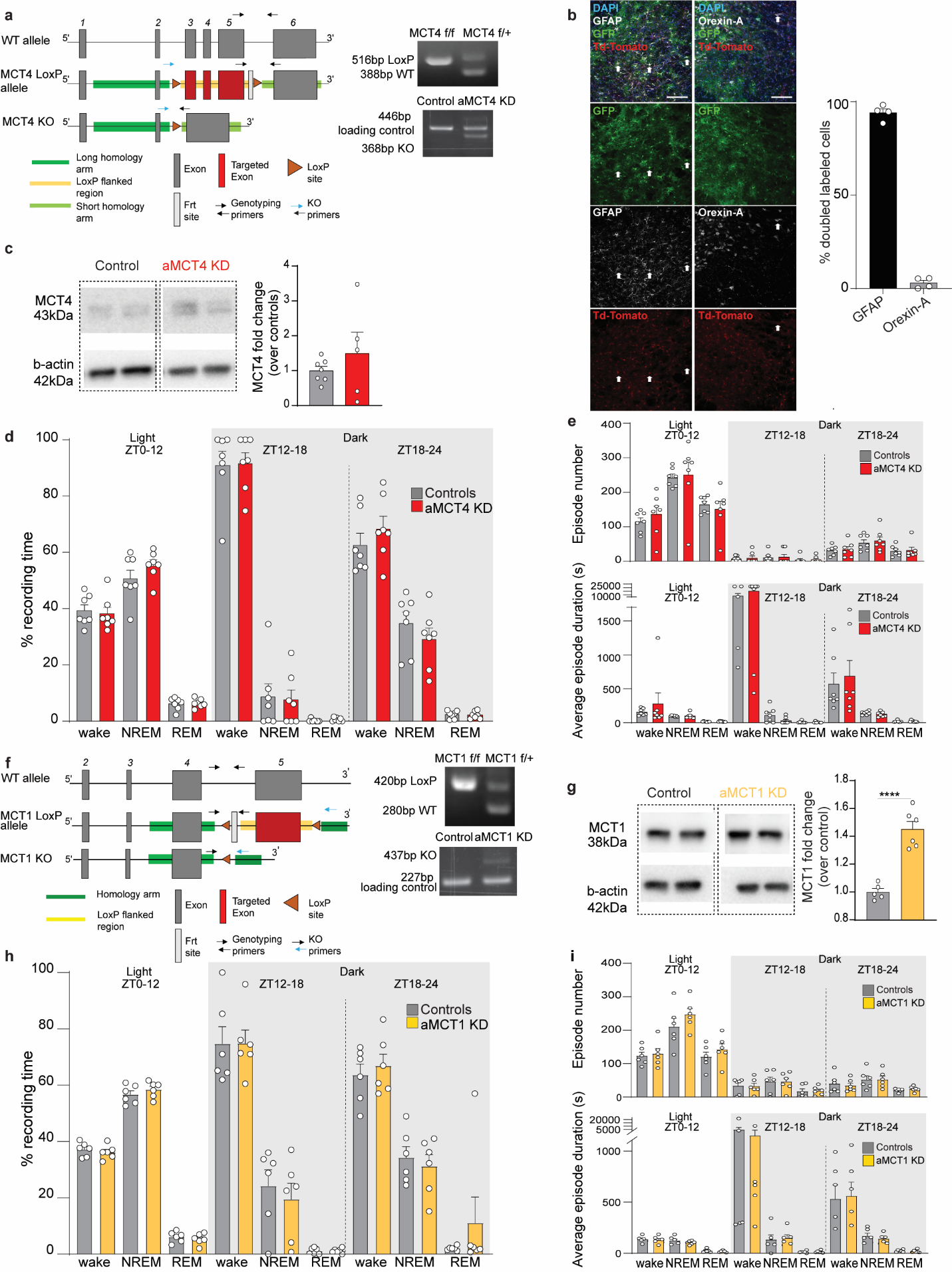


**Supplementary Fig. 2: Knockdown of astrocytic MCT4 or MCT1, 6 weeks after viral injections into the LH**

(a) Diagram of the MCT4 wild-type allele, LoxP flanked allele and KO allele (left panel). Right panels show genotype PCR for the floxed MCT4 allele in homozygous (MCT4 f/f/) and heterozygous (MCT4 f/+) mice and the genotyping results of aMCT4 KD mouse and control mouse.

(b) Representative immunofluorescence micrographs representing viral injections (PhP.eB-GFAP(0.7)-iCre-T2A-eGFP) into the LH in Ai14 mice. GFP in green, GFAP or Orexin-A in white and endogenous Td-Tomato expressed upon Cre recombination in red. White arrows indicate representative double positive astrocytes (expressing GFAP and Td-Tomato) or double positive orexinergic neurons (expressing Orexin-A and Td-Tomato). Scale bar=100µm. Quantification of cells expressing Td-Tomato and labeled with either GFAP or Orexin-A (n=4 mice) on the right.

(c) Representative western blot image of MCT4 expression and densitometric quantification 6 weeks after injections (Controls n=7, aMCT4 KD n=5). β-Actin was used as a loading control. The results are expressed as the mean values relative to controls (±SEM) (two-tailed unpaired t test).

(d and h) Quantification of the average percentage of time spent in wake, REM or NREM sleep during the 24 h recording phase in control mice (n=7) and aMCT4 KD mice (n=7) (d) or in aMCT1 KD mice (n=6) and corresponding controls (n=6), showing no differences between the groups (h) (two-tailed unpaired t-test).

(e and i) Average of the number (top panel) and duration (bottom panel) of wake, REM and NREM sleep episodes during the 24 h recording phase in control mice (n=7) and aMCT4 KD mice (n=7) (e) or in aMCT1 KD mice (n=7) and corresponding control (n=6) (i) (two-tailed unpaired t-test).

(f) Diagram of the MCT1 wild-type allele, LoxP flanked allele and KD allele (left panel). Right panels show genotype PCR for the floxed MCT1 allele in homozygous (MCT1 f/f/) and heterozygous (MCT1 f/+) mice and the genotyping results of aMCT1 KD mouse and control mouse.

(g) Representative western blot image of MCT1 expression and densitometric quantification 6 weeks after injections (Controls n=5, aMCT1 KD n=6). β-Actin was used as a loading control. The results are expressed as the mean values relative to controls (±SEM) (two-tailed unpaired t-test; ****p<0.0001). Source data are provided as a Source Data file.


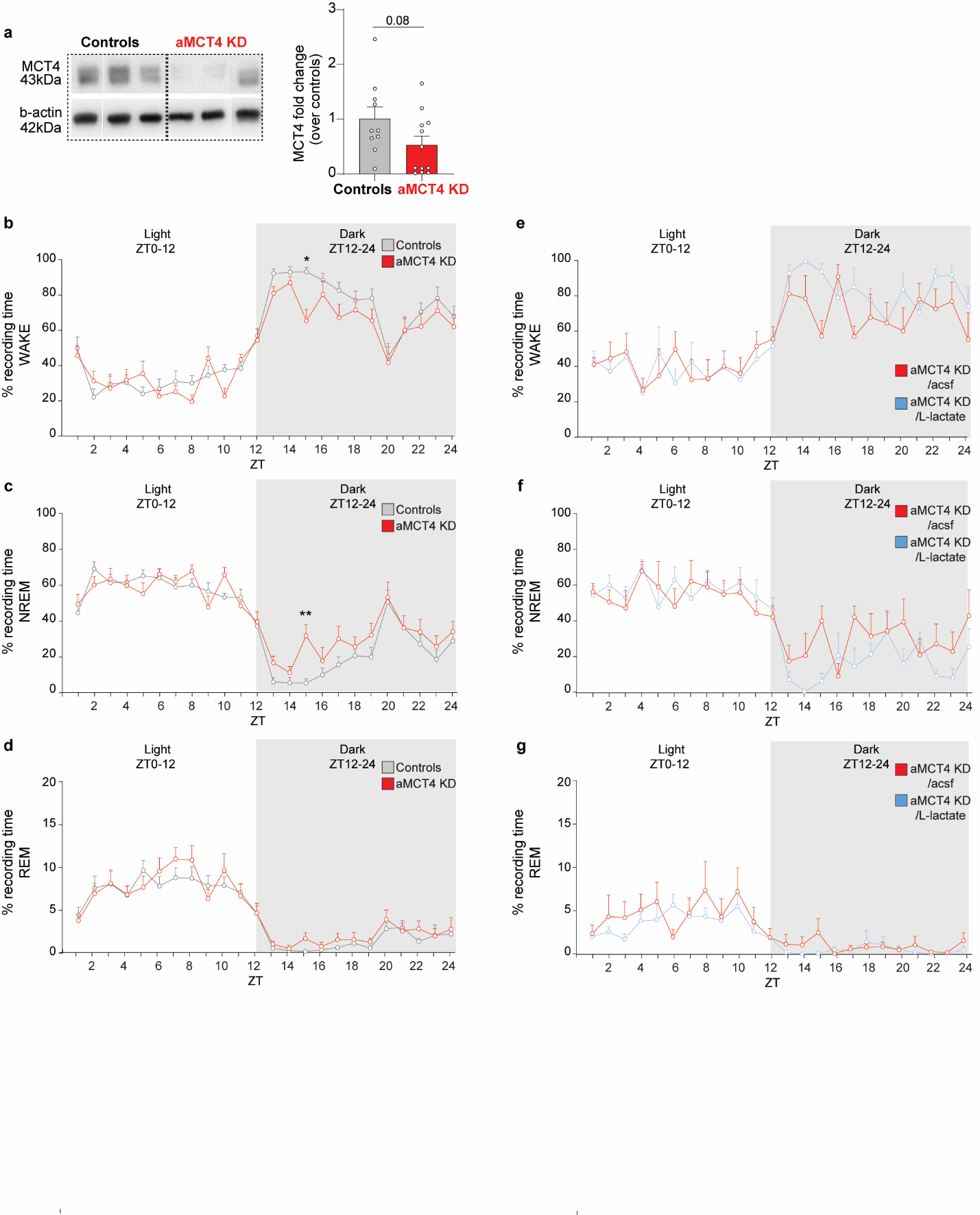


**Supplementary Fig. 3: 12 weeks post-viral injection, knockdown of astrocytic MCT4 alters sleep/wake architecture.**

(a) Representative western blot image of MCT4 expression and densitometric quantification (Controls n=10, aMCT4KD n=12). β-Actin was used as a loading control (two-tailed unpaired t test).

(b - d) Averaged percentage of the time spent in wake (b), NREM (c), and REM sleep (d) per hour in aMCT4 KD mice compared to control mice. (aMCT4 KD n=12; controls n=15, Two-Way ANOVA followed by Sidak post hoc test; %wake *p=0.010, %NREM **p=0.006)

(e – g) Averaged percentage of the time spent in wake (e), NREM (f), and REM sleep (g) per hour in aMCT4 KD mice upon lactate infusion. (a , b and c: n=5, Two-Way ANOVA followed by Sidak post hoc test). ZT= Zeitgeber Time. Pooled data are shown as mean ± SEM. Source data are provided as a Source Data file.


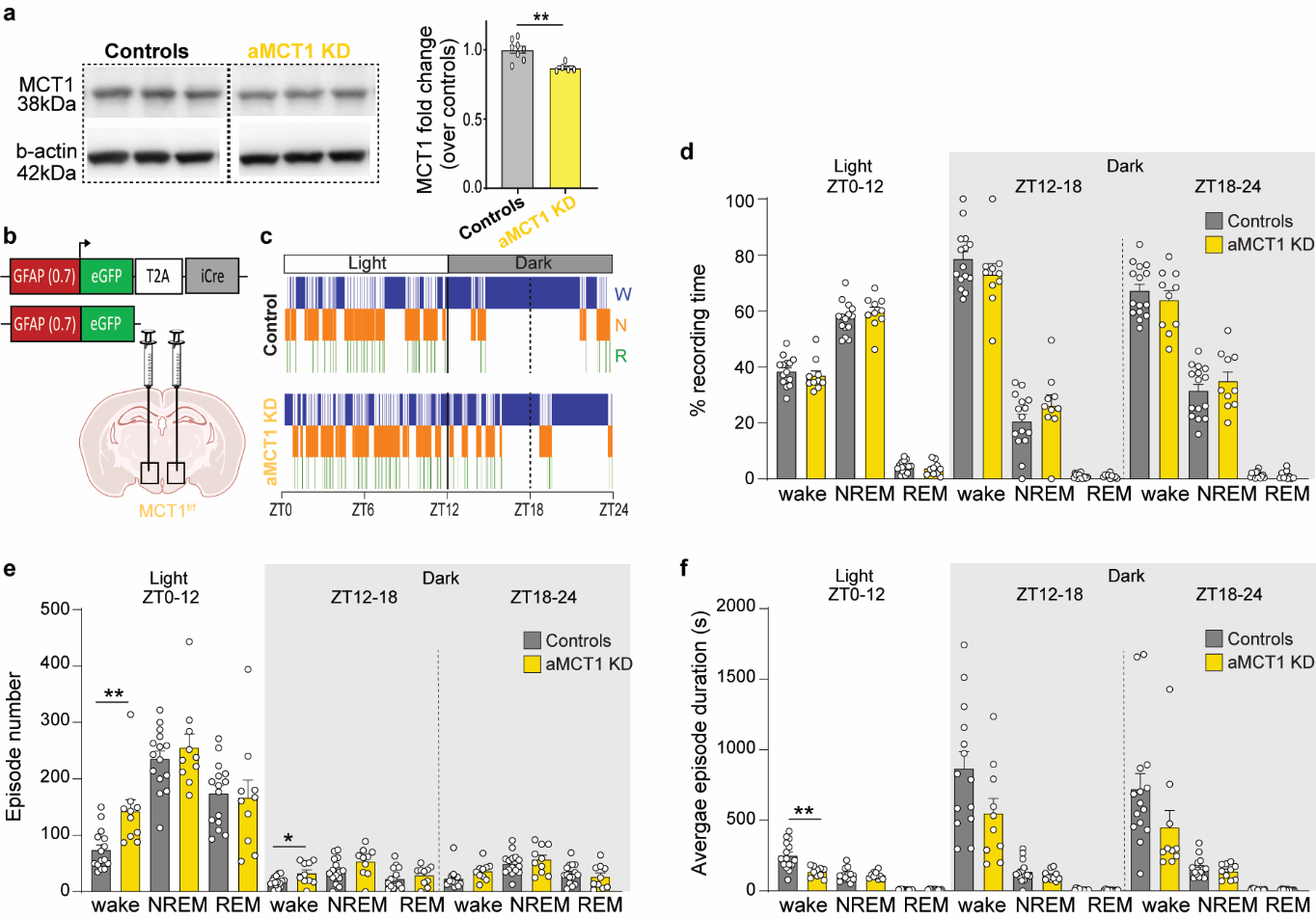


**Supplementary Fig. 4: Deletion of astrocytic MCT1 increases the number of wakefulness events during the dark phase**

(a) Representative western blot image of MCT1 expression and densitometric quantification 12 weeks after injection (Controls n=8, aMCT1 KD n=5). β-Actin was used as a loading control. The results are mean values relative to controls (±SEM). (two-tailed unpaired t test; **p=0.003).

(b) Schematic of stereotaxic injections of either Cre-encoding PhP.eB virus (aMCT1 KD mice) or control PhP.eB virus (control mice) in the LH of MCT1^f/f^ mice.

(c) Representative hypnograms of control mice and aMCT1 KD mice during 24 h EEG recordings (W – wake; N – NREM and R – REM).

(d) Quantification of the average percentage of time spent in wake, NREM or REM sleep during the 24h recording phase in aMCT1 KD mice compared to control mice.

(e) Average of the number of wake, NREM and REM episodes during the 24h recording phase in aMCT1 KD mice compared to control mice.

(f) Average duration of the wake, NREM and REM episodes during the 24h recording phase.

(aMCT1 KD n=10; controls n=15, two-tailed unpaired t test; ZT0-12 episode number wake **p=0.003, ZT12-18 episode number wake *p=0.012, ZT0-12 episode duration wake **p=0.002). Pooled data are shown as mean ± SEM. Source data are provided as a Source Data file.


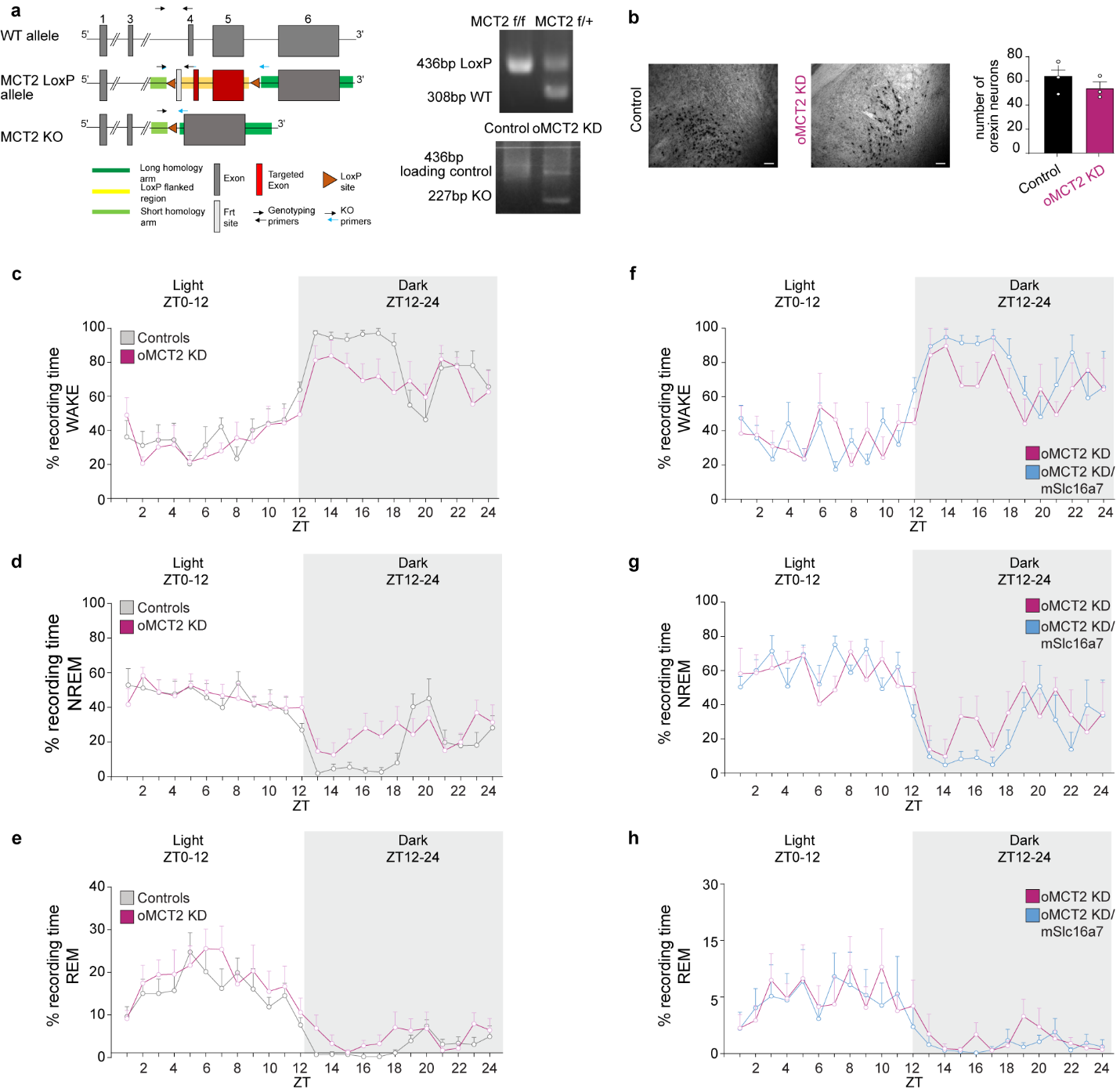


**Supplementary Figure 5: Orexinergic knockdown of MCT2**

(a) Diagram of the MCT2 wild-type allele, LoxP flanked allele and KDO allele (left panel). Right panels show genotype PCR for the floxed MCT2 allele in homozygous (MCT2 f/f/) and heterozygous (MCT2 f/+) mice and the genotyping results of oMCT2 KDO mouse and control mouse.

(b) Representative immunohistochemical micrographs representing Orexin-A expression in control mice (n=3) and oMCT2 KDO mice (n=3) and quantification showing no differences between groups (two-tailed unpaired t test). Scale bar = 50 µm.

(c - e) Averaged percentage of the time spent in wake (c), NREM (d), and REM sleep (e) per hour in oMCT2 KD mice compared to control mice. (Controls n=7, oMCT2 KD n=7, Two-Way ANOVA followed by Sidak post hoc test).

(f - h) Averaged percentage of the time spent in wake (f), NREM (g), and REM sleep (h) per hour in oMCT2 KD mice upon MCT2 re-expression. (n=5, Two-Way ANOVA followed by Sidak post hoc test). Source data are provided as a Source Data file.


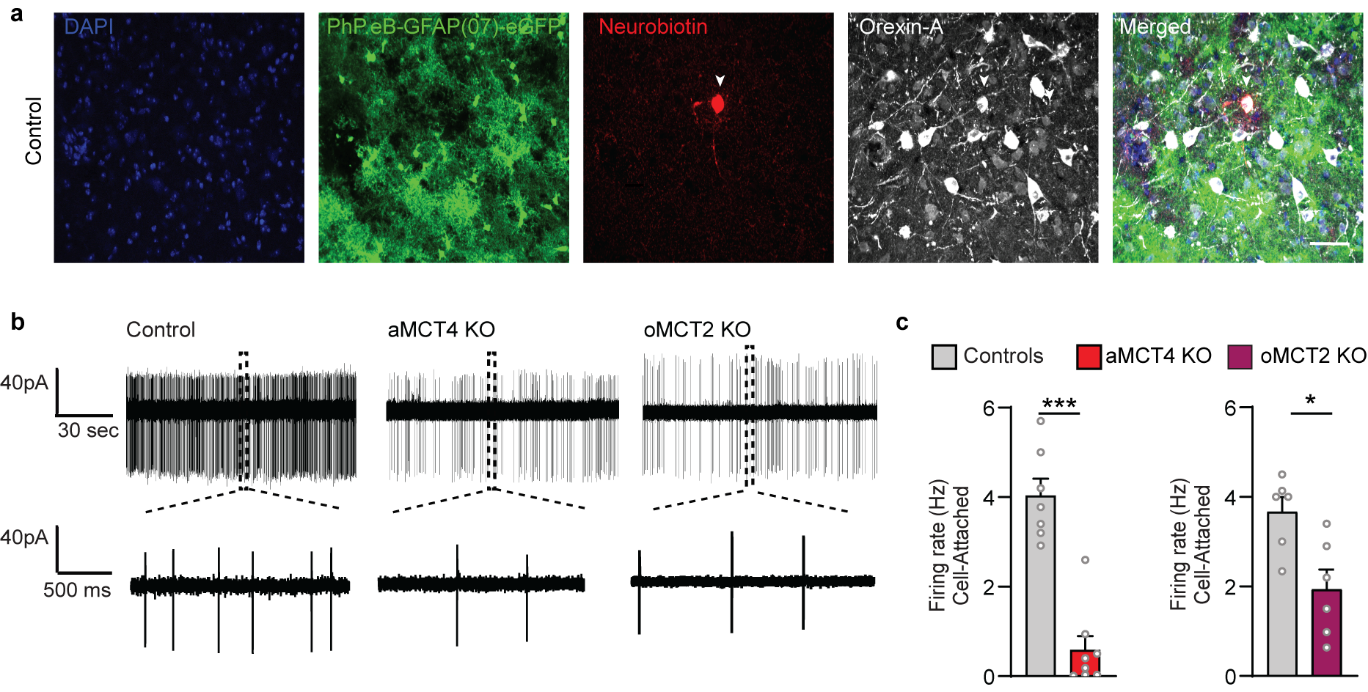


**Supplementary Fig. 6: Deletion of astrocytic MCT4 and orexinergic MCT2 decreases spontaneous tonic firing of orexinergic neurons.**

(a) Brain slice from a control mouse injected with PhPeB-GFAP(07)-eGFP into the LH showing Cre:GFP expression (green) in astrocytes. The image illustrates the Neurobiotin-filled neuron (red) colocalizing with the orexinergic marker (Orexin-A, white) indicated by the arrowhead. DAPI nuclear staining (blue). Scale bar, 20 µM.

(b) Representative cell-attached recordings in orexinergic neurons from control mice (left), aMCT4 KD mice (middle) and oMCT2 KD mice (right). The bottom traces show an expanded timescale of those recordings.

(c) Average firing rate in cell-attached mode confirms a decrease in activity in orexinergic neurons in aMCT4 KD (Controls, n=7 cells from four mice and aMCT4 KD, n=8 cells from five mice; two-tailed Mann‒Whitney test, ***p=0.0003) and oMCT2 KD (Controls, n= 6 cells from three mice and oMCT2 KD mice, n=6 cells from three mice; two-tailed Mann‒Whitney test, *p=0.0130). Pooled data are shown as mean ± SEM. Source data are provided as a Source Data file.


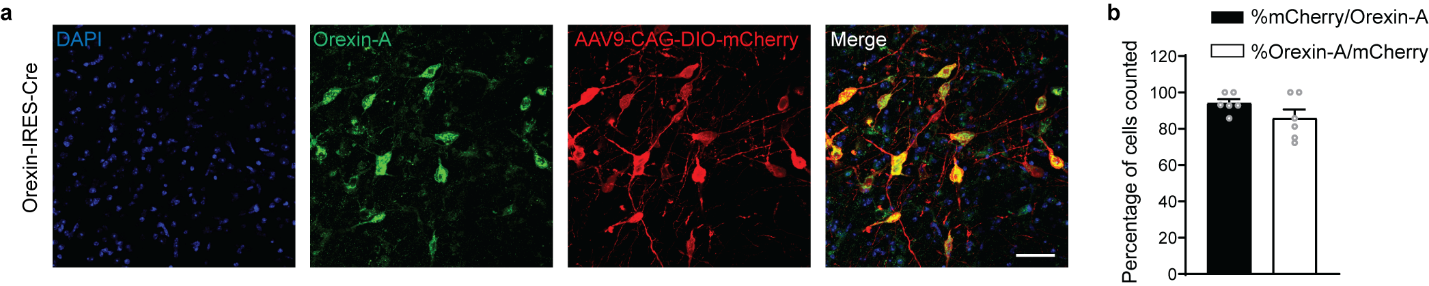


**Supplementary Fig. 7. Selective expression of Cre recombinase in orexinergic neurons in the LH.**

(a) Section of the LH from Orexin-IRES-Cre mouse stained with an antibody against orexin-A and DAPI (nuclear staining) 15 days after injection of AAV9-CAG-DIO-mCherry into the LH. Cre:mCherry-expressing cells (red) were positive for Orexin-A (green). Scale bar, 20 µM.

(b) Percentage of mCherry+ cells (expressing CRE) that are Orexin-A+ (orexinergic neurons) and percentage of Orexin-A+ cells that are mCherry+. n=6 brain sections from four Orexin-IRES-Cre mice. Pooled data are shown as mean ± SEM. Source data are provided as a Source Data file.


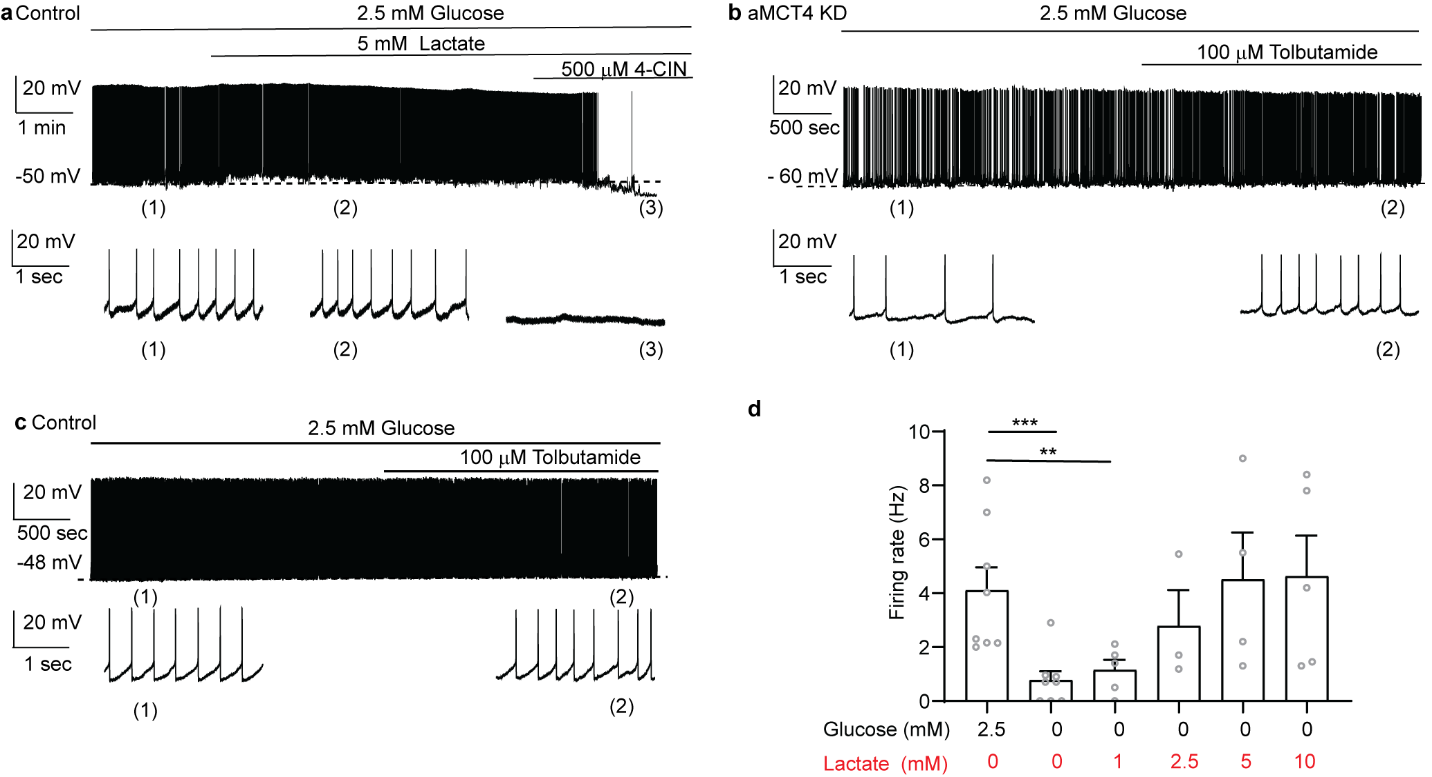


**Supplementary Fig. 8: Lactate does not increase the firing rate of orexin neurons in control mice.**

(a) A representative trace shows that lactate does not affect orexinergic activity in Control cells, while 4-CIN strongly hyperpolarizes and decreases firing in the same cell, even in the presence of extracellular lactate

(b-c) Tolbutamide increases the activity of an orexinergic neuron from aMCT4KO mice (b) but not in control cell (c).

(d) Whole-cell recordings, manipulating lactate concentrations (1, 2.5, 5, and 10 mM) in the absence of glucose, ([2.5 mM Glucose, 0 mM Lactate], n = 8 cells/5 mice; [0 mM Glucose, 0 mM Lactate], n= 8 cells/5 mice; [0 mM Glucose, 1 mM Lactate], n= 5 cells/5 mice; [0 mM Glucose, 2.5 mM Lactate], n= 3 cells/3 mice; [2.5 mM Glucose, 5 mM Lactate], n = 4 cells/4 mice; [0 mM Glucose, 10 mM Lactate], 5 cells/4 mice). Mixed-effects analysis followed by Dunnett’s multiple comparison test; ***p=0.0003, **p=0.0028.

Pooled data are shown as mean ± SEM. Source data are provided as a Source Data file.
