# Supplementary material for: Astrocytic metabolic control of orexinergic activity in the lateral hypothalamus regulates sleep and wake architecture
Source: Nat Commun. 2024 Jul 16;15:5979. doi: 10.1038/s41467-024-50166-7 (PMC11252394; doi:10.1038/s41467-024-50166-7)
Supplement: Supplementary file 3 — Reporting Summary [file 41467_2024_50166_MOESM3_ESM.pdf]

nature portfolio

Corresponding author(s): Martina Chiacchiaretta
Philip Haydon

Last updated by author(s): May 22, 2024

Reporting Summary

Nature Portfolio wishes to improve the reproducibility of the work that we publish. This form provides structure for consistency and transparency in reporting. For further information on Nature Portfolio policies, see our Editorial Policies and the Editorial Policy Checklist.

Statistics

For all statistical analyses, confirm that the following items are present in the figure legend, table legend, main text, or Methods section.

n/a	Confirmed	
☐	☒	The exact sample size (*n*) for each experimental group/condition, given as a discrete number and unit of measurement
☐	☒	A statement on whether measurements were taken from distinct samples or whether the same sample was measured repeatedly
☐	☒	The statistical test(s) used AND whether they are one- or two-sided<br>*Only common tests should be described solely by name; describe more complex techniques in the Methods section.*
☒	☐	A description of all covariates tested
☐	☒	A description of any assumptions or corrections, such as tests of normality and adjustment for multiple comparisons
☐	☒	A full description of the statistical parameters including central tendency (e.g. means) or other basic estimates (e.g. regression coefficient) AND variation (e.g. standard deviation) or associated estimates of uncertainty (e.g. confidence intervals)
☐	☒	For null hypothesis testing, the test statistic (e.g. *F*, *t*, *r*) with confidence intervals, effect sizes, degrees of freedom and *P* value noted<br>*Give P values as exact values whenever suitable.*
☒	☐	For Bayesian analysis, information on the choice of priors and Markov chain Monte Carlo settings
☒	☐	For hierarchical and complex designs, identification of the appropriate level for tests and full reporting of outcomes
☒	☐	Estimates of effect sizes (e.g. Cohen's *d*, Pearson's *r*), indicating how they were calculated

Our web collection on statistics for biologists contains articles on many of the points above.

Software and code

Policy information about availability of computer code

Data collection	Electrophysiology data were colleted using Clampex 9.2 and 10.6 from pClamp software (Molecular Device), EEG/EMG data and lactate recordings were acquired using Sirenia Acquisition 2.2.7 (Pinnacle); Western blot images and PCR images were acquired using ImageQuant LAS 4000 software (Fujifilm); immunofluorescence images were acquired using BZ-X700 Image Acquisition (Keyence BZ-X700) or NIS-Elements AR (Nikon).
Data analysis	Electrophysiology data were analyzed using Clampfit 9.2 and 10.6 from pClamp software (Molecular Devices). Western blot images and immunofluorescence images were analyzed using Fiji Software. Statistical analysis has been performed using Prism7 and Prism8 software (GraphPad Software, Inc). Lactate biosensor analysis was performed using MATLAB 2021b . EEG/EMG data were analyzed using SleepSign v. 3.0 (KISSEI COMTEC)

For manuscripts utilizing custom algorithms or software that are central to the research but not yet described in published literature, software must be made available to editors and reviewers. We strongly encourage code deposition in a community repository (e.g. GitHub). See the Nature Portfolio guidelines for submitting code & software for further information.

Data

Policy information about availability of data

All manuscripts must include a data availability statement. This statement should provide the following information, where applicable:

- Accession codes, unique identifiers, or web links for publicly available datasets
- A description of any restrictions on data availability
- For clinical datasets or third party data, please ensure that the statement adheres to our policy

code used is available at the following link: https://osf.io/fwru5/?view_only=48ee97f56e424b219f66ef3d9d7ed20d
Source data are provided with this paper as Source Data file.
Mean values, sample sizes, and related statistics for 24EEG analysis can be found in the Statistics Table (https://osf.io/fwru5/?view_only=48ee97f56e424b219f66ef3d9d7ed20d).

Research involving human participants, their data, or biological material

Policy information about studies with human participants or human data. See also policy information about sex, gender (identity/presentation), and sexual orientation and race, ethnicity and racism.

Reporting on sex and gender	N/A
Reporting on race, ethnicity, or other socially relevant groupings	N/A
Population characteristics	N/A
Recruitment	N/A
Ethics oversight	n/A

Note that full information on the approval of the study protocol must also be provided in the manuscript.

Field-specific reporting

Please select the one below that is the best fit for your research. If you are not sure, read the appropriate sections before making your selection.

- [x] Life sciences
- [ ] Behavioural & social sciences
- [ ] Ecological, evolutionary & environmental sciences

For a reference copy of the document with all sections, see nature.com/documents/nr-reporting-summary-flat.pdf

Life sciences study design

All studies must disclose on these points even when the disclosure is negative.

Sample size	No statistical methods were used to predetermine sample size. >3 mice were used for each experiment, and greater number of slices, based on our previous publication (Clasadonte et al., Neuron,2017)
Data exclusions	No data points were excluded from the study.
Replication	Multiple mice from independent cohorts were used for all experimental settings. The number of mice and cells used in each experiment is reported in every figure legend. Immunofluorescence images and Western blots were repeated in at least three animals.
Randomization	Mice were randomized for each litter. Equal number of mice for each litter were randomly assigned to control groups or Cre treated group.
Blinding	No blinding method was used. Analysis was performed using semi-automated softwares that reduced the risk of bias.

Reporting for specific materials, systems and methods

We require information from authors about some types of materials, experimental systems and methods used in many studies. Here, indicate whether each material, system or method listed is relevant to your study. If you are not sure if a list item applies to your research, read the appropriate section before selecting a response.

Materials & experimental systems

n/a	Involved in the study	
☐	☒	Antibodies
☒	☐	Eukaryotic cell lines
☒	☐	Palaeontology and archaeology
☐	☒	Animals and other organisms
☒	☐	Clinical data
☒	☐	Dual use research of concern
☒	☐	Plants

Methods

n/a	Involved in the study	
☒	☐	ChIP-seq
☒	☐	Flow cytometry
☒	☐	MRI-based neuroimaging

Antibodies

Antibodies used

All antibodies are commercially purchased:

Chicken polyclonal anti-GFAP Abcam Cat#AB4674; RRID: AB_304558; 1:500 dilution
Rabbit polyclonal anti-Orexin-A Millipore Cat#AB3704; RRID: AB_91545; 1:500 dilution
Rabbit polyclonal anti-Orexin-A Abcam Cat#AB6214; RRID: AB_305380; 1:500 dilution
Mouse monoclonal anti-Orexin-A R&D Systems Cat#MAB763; Clone # 97505; RRID: AB_2117627; 1:200 dilution
Rabbit polyclonal anti-mCherry Thermo Fisher Cat#600-401-P16; RRID: AB_2614470
Chicken polyclonal anti-GFP Abcam Cat#AB13970; RRID: AB_300798; 1:500 dilution
Rabbit polyclonal anti-MCT1 Proteintech Cat#20139-1-AP; RRID: AB_2878645; 1:10.000 dilution
Rabbit polyclonal anti-SLC16A3 Novus Biologicals Cat#NBP1-81251; RRID: AB_11033184; 1:1000 dilution
HRP-conjugated goat polyclonal anti-rabbit Thermo Fisher Cat#31460; RRID: AB_228341; 1:15.000 dilution
HRP-conjugated goat polyclonal anti-mouse Thermo Fischer Cat#31430; RRID: AB_228307; 1:15.000 dilution
Mouse monoclonal anti-β-Actin Sigma Cat#A1978; clone AC-15; RRID: AB_476692; 1:1000 dilution
Goat polyclonal anti-rabbit, Alexa Fluor 633 conjugated Invitrogen Cat#A21071; RRID: AB_2535732; 1:1000 dilution
Goat polyclonal anti-chicken, Alexa Fluor 633 conjugated Invitrogen Cat#A21103; RRID: AB_2535756; 1:1000 dilution
Goat polyclonal anti-rabbit, Alexa Fluor 546 conjugated Invitrogen Cat#A11035; RRID: AB_2534093; 1:1000 dilution
Goat polyclonal anti-chicken, Alexa Fluor 488 conjugated Invitrogen Cat#A11039; RRID: AB_2534096; 1:1000 dilution
Goat polyclonal anti-rabbit, Biotin Conjugate Abcam Cat#AB6720; RRID: AB_954902; 1:500 dilution
Goat polyclonal anti-mouse, Alexa Fluor 488 conjugated Invitrogen, Cat#A-11001; RRID: AB_2534069; 1:1000 dilution

Validation

Primary antibodies were validated by the manufacturer and confirmed by specific labeling of target molecules:

-Chicken polyclonal anti-GFAP Abcam (https://www.abcam.com/products/primary-antibodies/gfap-antibody-ab4674.html)
-Rabbit polyclonal anti-Orexin-A Millipore (https://www.emdmillipore.com/US/en/product/Anti-Orexin-A-Antibody,MM_NF-AB3704?ReferrerURL=https%3A%2F%2Fwww.google.com%2F&bd=1)
-Rabbit polyclonal anti-Orexin-A Abcam (https://www.abcam.com/products/primary-antibodies/orexin-a-antibody-ab6214.html)
-Mouse monoclonal anti-Orexin-A R&D Systems (https://www.rndsystems.com/products/human-mouse-orexin-a-hypocretin-1-antibody-97505_mab763)
-Rabbit polyclonal anti-mCherry Thermo Fisher (https://www.thermofisher.com/antibody/product/mCherry-Antibody-Polyclonal/600-401-P16)
-Chicken polyclonal anti-GFP Abcam (https://www.abcam.com/en-no/products/primary-antibodies/gfp-antibody-ab13970)
-Rabbit polyclonal anti-MCT1 Proteintech (https://www.ptglab.com/products/MCT1-Antibody-20139-1-AP.htm)
- Rabbit anti-SLC16A3 Novus Biologicals (https://www.novusbio.com/products/slc16a3-antibody_nbp1-81251)
-HRP-conjugated goat polyclonal anti-rabbit Thermo Fisher (https://www.thermofisher.com/antibody/product/Goat-anti-Rabbit-IgG-H-L-Secondary-Antibody-Polyclonal/31460)
-HRP-conjugated goat polyclonal anti-mouse Thermo Fischer(https://www.thermofisher.com/antibody/product/Goat-anti-Mouse-IgG-H-L-Secondary-Antibody-Polyclonal/31430)
-Mouse monoclonal anti-β-Actin Sigma (https://www.sigmaaldrich.com/US/en/product/sigma/a1978)
-Goat polyclonal anti-rabbit, Alexa Fluor 633 conjugated Invitrogen(https://www.thermofisher.com/antibody/product/Goat-anti-Rabbit-IgG-H-L-Highly-Cross-Adsorbed-Secondary-Antibody-Polyclonal/A-21071)
-Goat polyclonal anti-chicken, Alexa Fluor 633 conjugated Invitrogen (https://www.thermofisher.com/antibody/product/Goat-anti-Chicken-IgY-H-L-Cross-Adsorbed-Secondary-Antibody-Polyclonal/A-21103)
-Goat polyclonal anti-rabbit, Alexa Fluor 546 conjugated Invitrogen (https://www.thermofisher.com/antibody/product/Goat-anti-Rabbit-IgG-H-L-Highly-Cross-Adsorbed-Secondary-Antibody-Polyclonal/A-11035)
-Goat polyclonal anti-chicken, Alexa Fluor 488 conjugated Invitrogen (https://www.thermofisher.com/antibody/product/Goat-anti-Chicken-IgY-H-L-Secondary-Antibody-Polyclonal/A-11039)
-Goat polyclonal anti-rabbit, Biotin Conjugate Abcam (https://www.abcam.com/products/secondary-antibodies/goat-rabbit-igg-hl-biotin-ab6720.html)
-Goat polyclonal anti-mouse, Alexa Fluor 488 conjugated Invitrogen (https://www.thermofisher.com/antibody/product/Goat-anti-Mouse-IgG-H-L-Cross-Adsorbed-Secondary-Antibody-Polyclonal/A-11001)

Animals and other research organisms

Policy information about studies involving animals; ARRIVE guidelines recommended for reporting animal research, and Sex and Gender in Research

Laboratory animals	Male and female mice were bred and housed on a 12/12 light/dark cycle, given standard chow and water ad libitum. Ambient temperature was maintained at 23 ± 1°C, and humidity was kept within the range of 40% to 60%.<br>MCT1 floxed mice, MCT4 floxed mice, MCT2 floxed mice, Orexin-IRES-Cre mice, Ai14 mice, C57Bl/6 mice have been used in this study. All transgenic lines have a C57Bl/6 background.<br>Homozygous MCT4floxed/floxed, MCT2 floxed/floxed and MCT1 floxed/floxed mice originated from Dr. L. Pellerin's lab (Department of Physiology, University of Lausanne, Lausanne, Switzerland). Upon receival, mice were cleared from quarantine, backcrossed with C57BL/6 mice, and bred in our facility.<br>Orexin-IRES-Cre mice were generated, validated, and kindly provided by Dr. Dong Kong and crossed with MCT2 floxed/floxed mice.<br>Ai14 mice (#007909, Jackson Laboratory) have been kindly donated from Dr. Yongjie Yang.<br>oMCT2 KO, Ai14 and control mice were used at 7 weeks old. aMCT4KO, aMCT1 KO and control mice were used at 4 weeks and 12 weeks post viral injection. Adeno-virus vector stereotaxic injection was performed in 7 weeks old mice.
Wild animals	This study did not involve wild animals.
Reporting on sex	Both female and male mice have been equally used for collecting data. No sex-biased analysis has been carried out, nor has the analysis been disaggregated by sex. Preclinical studies investigating the role of orexinergic neurons in the regulation of sleep and wakefulness in rodents did not show any sex-based differences, ultimately supporting the combination of both sexes. Based on these findings and the aim to reduce the number of animals used whenever feasible, we combined female and male mice in our experimental settings.
Field-collected samples	The study did not involve samples collected from the field.
Ethics oversight	All animal protocols were approved by the Tufts Institutional Animal Care and Use Committee.

Note that full information on the approval of the study protocol must also be provided in the manuscript.

Plants

Seed stocks	N/A
Novel plant genotypes	N/A
Authentication	N/A
